# Mapping of Genetic Locus for Leaf Trichome Formation in Chinese Cabbage Based on Bulked Segregant Analysis

**DOI:** 10.3390/plants10040771

**Published:** 2021-04-14

**Authors:** Rujia Zhang, Yiming Ren, Huiyuan Wu, Yu Yang, Mengguo Yuan, Haonan Liang, Changwei Zhang

**Affiliations:** State Key Laboratory of Crop Genetics and Germplasm Enhancement, College of Horticulture, Nanjing Agricultural University, Nanjing 210095, China; 2018204020@njau.edu.cn (R.Z.); 2018104075@njau.edu.cn (Y.R.); 2019104075@njau.edu.cn (H.W.); 2019804226@njau.edu.cn (Y.Y.); 2020104077@njau.edu.cn (M.Y.); 2020804243@njau.edu.cn (H.L.)

**Keywords:** bulked segregant analysis, Chinese cabbage, leaf trichome

## Abstract

Chinese cabbage is a leafy vegetable, and its leaves are the main edible organs. The formation of trichomes on the leaves can significantly affect its taste, so studying this phenomenon is of great significance for improving the quality of Chinese cabbage. In this study, two varieties of Chinese cabbage, W30 with trichome leaves and 082 with glabrous leaves, were crossed to generate F_1_ and F_1_ plants, which were self-fertilized to develop segregating populations with trichome or glabrous morphotypes. The two bulks of the different segregating populations were used to conduct bulked segregant analysis (BSA). A total of 293.4 M clean reads were generated from the samples, and plants from the trichome leaves (AL) bulk and glabrous leaves (GL) bulk were identified. Between the two DNA pools generated from the trichome and glabrous plants, 55,048 SNPs and 272 indels were generated. In this study, three regions (on chromosomes 6, 10 and scaffold000100) were identified, and the annotation revealed three candidate genes that may participate in the formation of leaf trichomes. These findings suggest that the three genes—*Bra025087* encoding a cyclin family protein, *Bra035000* encoding an ATP-binding protein/kinase/protein kinase/protein serine/threonine kinase and *Bra033370* encoding a WD-40 repeat family protein–influence the formation of trichomes by participating in trichome morphogenesis (GO: 0010090). These results demonstrate that BSA can be used to map genes associated with traits and provide new insights into the molecular mechanism of leafy trichome formation in Chinese cabbage.

## 1. Introduction

Chinese cabbage (*Brassica rapa* ssp. *pekinensis*), one of the subspecies of *Brassica rapa* [1], is one of the most popular vegetable crops in Asia, and its leaves are the main edible organs. Trichome formation in Chinese cabbage during its growth and development may affect how it tastes to consumers. The formation of leaves’ trichomes is receiving increasing attention from researchers, but the literature on its molecular mechanism remains insufficient.

Trichomes play important roles in water regulation, temperature control and the protection of plants against biotic and abiotic stresses, thereby increasing their tolerance to changes in the environment [2]. However, if the edible part is covered with trichomes, it may influence the appearance and mouthfeel [3,4]. This has caused researchers to attach greater importance to the molecular mechanism of the formation of trichomes. Trichomes arise at an early stage of organ morphogenesis out of the epidermal progenitor cells that also give rise to other cell types such as stomata and pavement cells. Trichomes are single-celled and hairy structures that develop on the epidermis of the aerial parts, including leaves, stems, fruits and sepals. Previous studies have reported the molecular mechanism of trichome formation in other plants, such as Arabidopsis [5], rice [6], cotton [7], cucumber [8], tomato [9] and maize [10]. However, few studies have been reported on trichome formation in Chinese cabbage [11].

Bulked segregant analysis (BSA) is a simple and rapid approach that uses segregating populations to identify molecular markers that are tightly linked to the causal gene underlying a given phenotype [12,13]. Two bulked pools with extreme traits and two parental lines were constructed for high-throughput next-generation sequencing (NGS) to identify polymorphic markers and conduct correlation analysis. The aim of the latter is to annotate the functions of the genes in the mapped regions by aligning the reference genome sequence of the species. With the development of DNA sequencing technology, NGS-based BSA has been used to map important genes in many plants, such as the yellow rind formation gene in watermelon [14], the branching habit trait in cultivated peanut [15], a candidate nicosulfuron sensitivity gene in maize [16], grain-shape-related loci in rice [17] and genes associated with the heading type of Chinese cabbage [18]. A large number of studies have proven that bulked segregant analysis is a reliable method for identifying loci associated with traits in plants.

In this study, we applied BSA-Seq to identify the pathways and genes related to the formation of trichomes. New SNPs and indels were developed to perform fine-linkage mapping of the previously located region. Taken together, the results provide new insights into the fine-mapping and identification of candidate genes in horticultural crops.

## 2. Results

### 2.1. Morphology and Genetic Analysis of Hairy and Hairless Chinese Cabbage Plant

**Figure 1:**
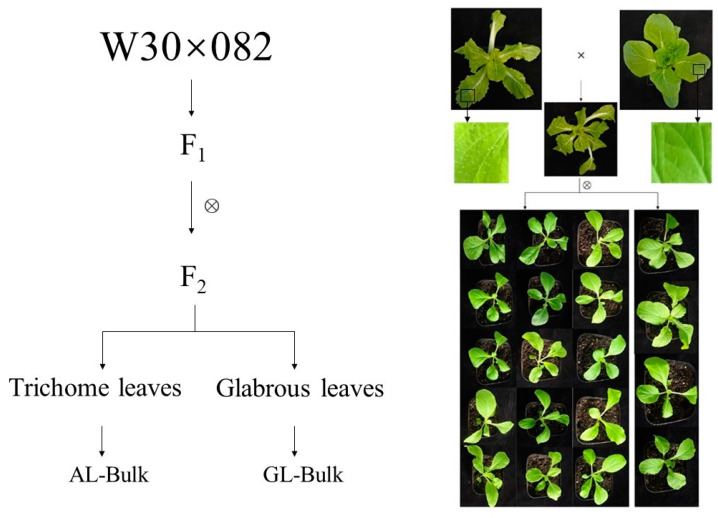
The phenotypes of plants of the W30 and 082 parental lines and their F_1_ and F_2_ populations. W30 had trichome leaves, and 082 had glabrous leaves. All F1 plants had trichomes and the leaves of the F2 population were classified as either trichome or glabrous.

There were significant differences in the surfaces of leaves between the W30 and 082 varieties of Chinese cabbage, which was further confirmed by integrated microscopy. F_1_ plants from a cross between W30 and 082 displayed trichome leaves. When the F_1_ plants were self-fertilized, the F_2_ plants showed different phenotypes: some had trichome leaves, and others had glabrous leaves (Figure 1). After sowing the F_2_ generation, a total of 294 plants were grown to observe whether the leaves formed trichomes. Among these plants, 212 developed glandular trichomes, while 82 plants were observed with glabrous leaves. This corresponds to a three to one segregation ratio (Table 1). These results demonstrate that trichome formation in Chinese cabbage is controlled by a dominant gene.

### 2.2. Construction and Sequencing of the Trichome Leaves (AL) Bulk and Glabrous Leaves (GL) Bulk Samples and Parental Lines

In order to further explore the candidate genes that regulate the formation of trichomes, we used the BSA-Seq strategy to identify candidate regions and find genes related to the formation of trichomes. Fifty plants with glandular trichomes and fifty plants with glabrous leaves were randomly selected from the F_2_ population, which contained a mixture of the AL-bulk and GL-bulk, and sequenced with their parental lines. After screening to remove low-quality reads, the two parents were resequenced, resulting in 37,948,696 and 36,842,550 reads and 11.38 and 11.05 Gb from W30 and 082, respectively. A total of 65.57 Gb clean data were obtained from the two bulks (33.06 Gb for the AL-bulk and 32.51 Gb for the GL-bulk). After mapping these reads to the reference genome, the coverage of AL and GL genomes was found to be 78× and 76×, respectively (Table 2).

### 2.3. Selection of Candidate Regions

**Figure 2:**
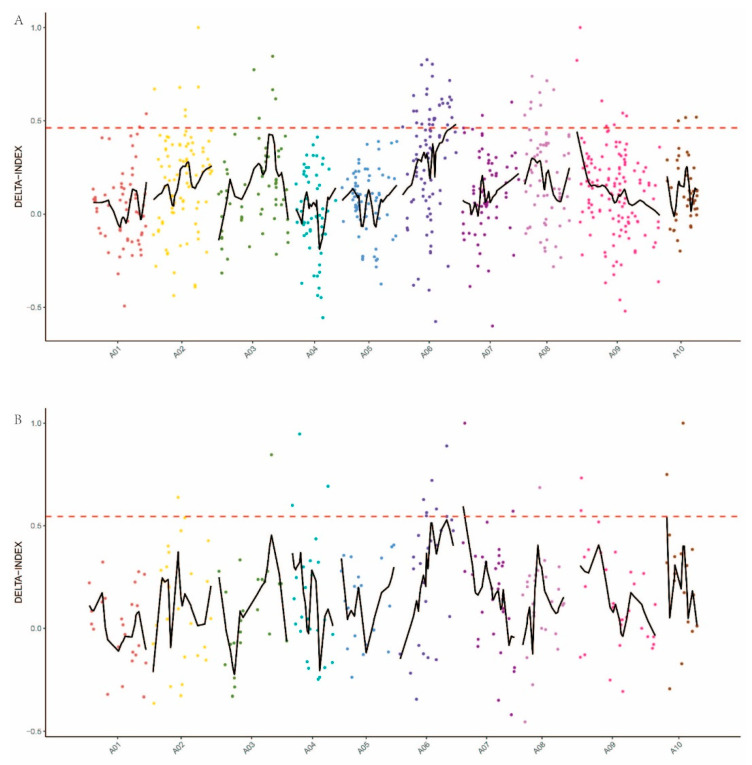
Candidate genomic regions for trichome formation identified using (**A**) the SNP-index algorithm and (**B**) the indel-index algorithm. The red dashed horizontal lines represent the threshold for defining a significant association. The *X*-axis shows the chromosome position and the different colors represent the different chromosomes. The *Y*-axis represents the Δ-index values.

Between AL and GL bulks, 3581 SNP sites were screened according to the principle of genotypic inconsistency (inconsistency between samples or heterozygous SNP sites) in the progeny mixing pool (AL and GL), and the depth was no less than 5X. To identify the genomic region associated with the trichome formation, we used the SNP-index to measure the allele segregation of SNPs between the two bulks. The method used to calculate the Δ(SNP-index) was in accordance with Yuanting [19]. In the method for determining the Δ(SNP-index) threshold of the nonreference genome, a Δ(SNP-index) greater than 0.99 was selected as the threshold for defining significant associations of a marker with traits; that is, a marker larger than the threshold was deemed to be significantly associated with traits. Six significant regions associated with trichome formation were detected by Δ(SNP-index) analysis (Figure 2A). They were located on Scaffold000100 from 160,071 to 260,071, Scaffold001011 from −49,220 to 50,780, Scaffold004266 from −49,807 to 50,193, Scaffold000169 from 132,175 to 132,181 and chromosome 6 from 22,044,767 to 22,246,745 and 23,704,762 to 24,097,454 bp. The identified regions contain a total of 894,682 bp and 110 genes were contained (Table 3).

According to the principle of genotype inconsistency (inconsistency between samples or heterozygous indel sites) in the progeny mixing pool (AL and GL), the depth was no less than 5X, and 1131 indel sites were screened. Using the indel data, three candidate regions were identified (Figure 2B). These regions are located on chromosome 7 from 1,892,096 to 1,992,096, chromosome 10 from 2,673,013 to 2,773,013 and scaffold000100 from 764,907 to 864,907. These candidate regions have a total length of 30,003 bp and contain a total of 45 genes (Table 4).

### 2.4. Gene Ontology (GO) Classification Analysis of Candidate Genes

**Figure 3:**
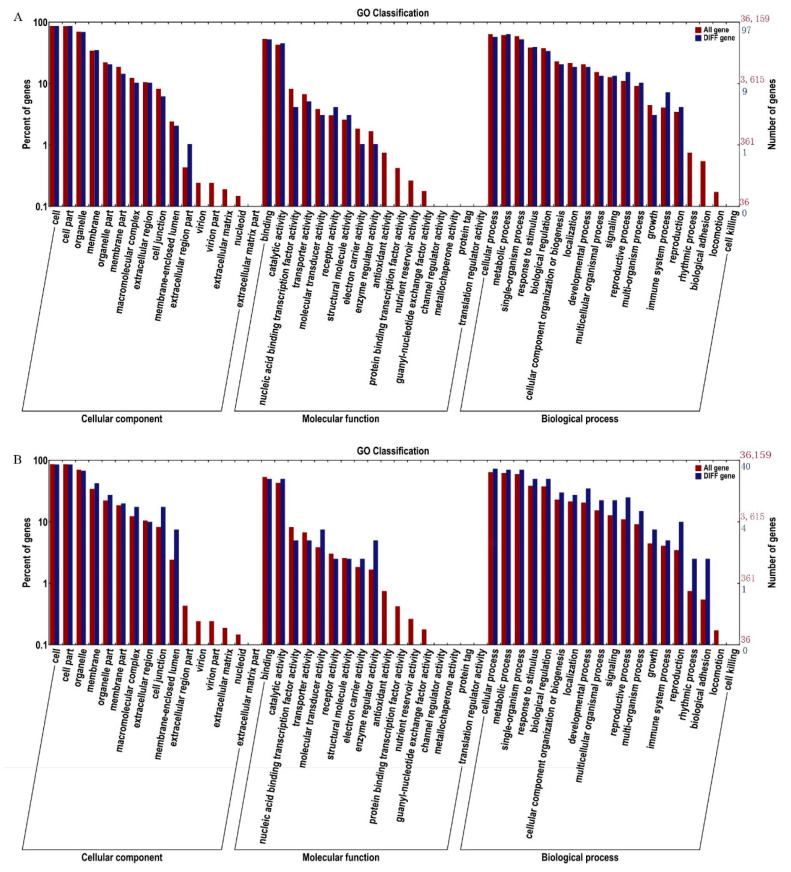
Functional enrichment analysis based on candidate genes GO classification for the (**A**) SNP-selected region and (**B**) the indel-selected region.

A GO classification analysis was carried out to understand the functions of all the candidate genes identified in the association analysis of SNPs and small indels. Comparing the AL-bulk with the GL-bulk revealed a total of 108 candidate genes in the analysis of SNPs, and 44 candidate genes were identified by analyzing the effects of small indels through GO annotation (Figure 3). All candidate genes are divided into three categories: biological processes, cellular components, and molecular functions. In general, the genes in candidate regions are more abundant in biological process classes than all other genetic background classes, including the other two categories identified in the analysis. Biological processes include cellular processes, metabolic processes, single-cell processes, response to stimulus, biological regulation, etc., indicating that the formation of leaf trichomes may affect the development and biological processes (Tables S1 and S2).

### 2.5. Candidate Genes for Hairiness

**Figure 4:**
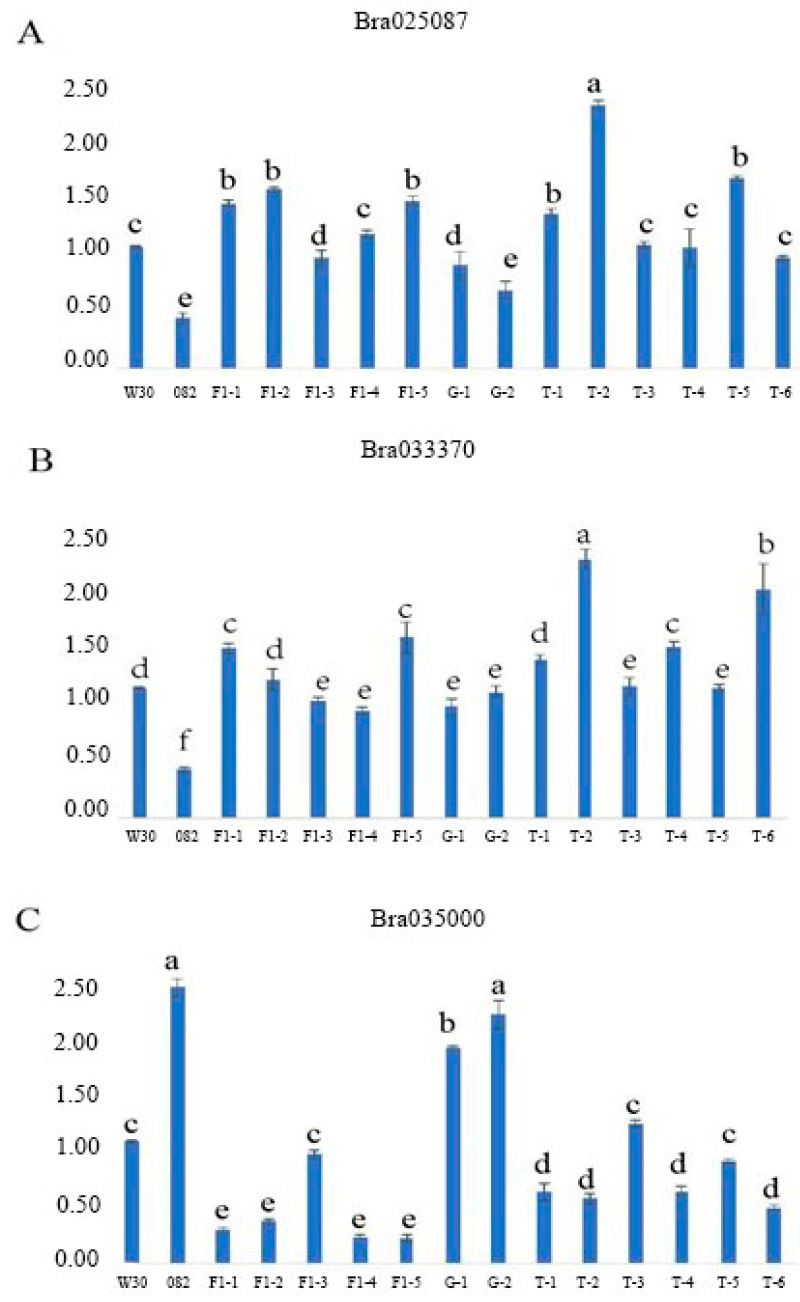
qRT-PCR expression analysis of the candidate genes in the parental lines and F_1_ and F_2_ generations. (**A**) *Bra025087*; (**B**) *Bra033370*; (**C**) *Bra035000*. W30: female parent; 082: male parent; F_1_: 082 × W30; G: F_2_ with glabrous leaves, T: F_2_ with trichome leaves. The data are the mean + SD; the letters above the bars indicate the significant differences determined by Tukey’s honestly significant difference method (*p* < 0.05).

Three candidate genes (*Bra025087*, *Bra035000* and *Bra033370*) that may be associated with leaf trichome formation in Chinese cabbage were identified through functional annotation (Tables S1 and S2). Table 5 shows the functional annotation of the three candidate genes. To analyze the functions of the candidate genes in the leaf trichome formation, qRT-PCR analysis was performed on W30, 082, five F_1_ plants, two F_2_ plants with glabrous leaves and six F_2_ plants with trichome leaves. According to the results of qRT-PCR analysis, the expression levels of two genes (*Bra025087* and *Bra033370*) were significantly higher in W30, F_1_ and F_2_ with leaf trichomes than in 082 and F_2_ with glabrous leaves (Figure 4A,B). On the contrary, the expression level of *Bra035000* was lower in W30, F_1_ and F_2_ with leaf trichomes than in 082 and F_2_ with glabrous leaves (Figure 4C). This indicates that the two genes (*Bra025087* and *Bra033370*) may facilitate the formation of leaf trichomes, whereas *Bra035000* may suppress it.

## 3. Discussion

Although there are many methods for analyzing trait-gene association analysis [20,21,22], BSA-Seq is still one of the most popular methods and used extensively for various taxa [18,23,24,25,26]. The greatest advantage of BSA-Seq is its simplicity in terms of both sample collection and data analysis. Bulked segregant RNA-Seq (BSR-Seq), which combines RNA-Seq with BSA-Seq, is an efficient method for reducing genome complexity [27]. However, if RNA-Seq is not performed using the right tissue at the appropriate developmental stage, the results of BSR-Seq can be misleading. In contrast, samples for BSA-Seq can be collected at any developmental stage and from any tissue. In addition, there is a high degree of structural variation within *Brassica rapa*, which will have a greater negative influence on the data analysis of BSR-Seq.

The key goal of BSA-Seq is to define the smallest candidate regions that are associated with the phenotype. Here, the candidate regions discovered by calculating the Δ(SNP-index) have a total length of 30,003 bp and contain a total of 45 genes. The three candidate genes (*Bra025087*, *Bra035000* and *Bra033370*) selected from the candidate regions are associated with leaf trichome formation in Chinese cabbage, as determined by gene functional annotation [28]. The candidate gene *Bra025087* is an ortholog of Arabidopsis CYCT 1;5 (*At5g45190*). The protein sequence of *Bra025087* exhibits high homology with that of AtCYCT1; 5 (Figure S1). CYCT 1;5 is a cyclin gene and classified as a T-type cyclin [29,30]. Arabidopsis contains five genes encoding cyclin T-like proteins (CYCT1;1 to CYCT1;5). In Arabidopsis, the expression of *CYCT1;5* in the anther and the inflorescence is slightly higher than that in other tissues [31]. It has been confirmed that CYCT1;5 induces complete resistance to CaMV, as well as altered leaf and flower growth, and delayed flowering. The article also reported that the adaxial trichomes of *CYCT1;5* RNAi plant leaves have two branches instead of the three branches found in typical trichomes of wild-type leaves [32]. The results of the RT-qPCR suggest that the expression of *Bra025087* is lower in all plants with glabrous leaves (Figure 4A). It is rational to speculate that the phenotype of *BrCYCT1;5* mutant is consistent with *AtCYCT1;5* RNAi. *BrCYCT1;5* (*Bra025087*) might positively regulate the growth and development of leaf trichome branches. The candidate gene *Bra035000* is an ortholog of Arabidopsis NIMA-related kinase 6 (NEK6, *At3G44200*), which regulates microtubule organization during anisotropic cell expansion. The similarity percentage between the protein sequences of Bra035000 and AtNEK6 is 80.41% (Figure S2). Previous studies have shown that Arabidopsis NEK6 regulates directional cell expansion through the depolymerization of cortical microtubules during interphase [33,34,35,36,37]. Takatani et al. found that NEK6-1 mutants exhibited wavy growth patterns in the fast-growing region of the hypocotyl, and their hypocotyls did not grow straight [38]. Our research also shows that plants with leaf trichomes have higher expression levels in all three generations (Figure 4B). *NEK6* was shown to be involved in the negative regulation of cell differentiation, further suppressing the development of leaf trichomes. *Bra033370*, another candidate gene, is an ortholog of *Arabidopsis SPI (At1g03060*), and its protein sequence shares 90.98% similarity with that of *At1g03060* (Figure S3). A decrease in the complexity of epidermal pavement cells and curled trichomes was observed in *SPI* mutants of Arabidopsis [39,40]. Thus, AtSPI positively regulates the normal growth of trichomes in Arabidopsis. *Arabidopsis thaliana*, as a fully sequenced model organism, is an excellent model system for studying cell differentiation in structures such as trichomes [41]. However, there is almost no related research on the three candidate genes in *Brassica rapa*. Therefore, the Arabidopsis model for studying trichomes can serve as a suitable reference for this process in *Brassica rapa*. However, we do not yet clearly know how the three candidate genes work in *Brassica rapa*, and further studies are needed.

## 4. Materials and Methods

### 4.1. Plant Materials and Phenotyping for Trichomes

W30, with trichome leaves, and nonheading Chinese cabbage 082 (082), with glabrous leaves, were obtained from the Chinese Academy of Agricultural Sciences of Nanjing Agricultural University. W30 and 082 were crossed, and confirmed F1 plants were self-fertilized to develop segregating populations. Then, 294 seeds of F2 were grown on the experimental farm of Nanjing Agricultural University in September of 2017. Two months later, the surviving plants displayed two contrasting trichome phenotypes with trichome (212) and glabrous (82) leaves. From these, we selected 50 plants of each morphotype and used them to establish R01 and R02 bulks for BSA-Seq analysis (Figure 1). Individual W30 and 082 plants were used to establish parental pools, and BSA-Seq was performed on three plants from each parent.

### 4.2. BSA-Seq and Sequence Alignment

A total of 100 plants (50 with trichome leaves and 50 with glabrous leaves) were selected from the F2 population for bulking. Two DNA pools were constructed by mixing equal amounts of DNA from 50 trichome leaves (AL-pool) and 50 glabrous leaves (GL-pool). DNA samples from the two bulks and two parental lines were prepared according to the standard Illumina protocol to construct sequencing libraries, which were sequenced on an Illumina HiSeq™2500 platform (Illumina, San Diego, CA, USA). Illumina Casava 1.8 was used for cleaning and filtering reads [42]. After low-quality and short reads were filtered out, the filtered short reads of each pool were mapped onto the Chinese cabbage reference genome sequence V1.5 (http://brassicadb.org/brad/ accessed on 30 March 2018) by BWA [43]. SNP calling was followed by GATK Best-Practices [44]. Both GATK and SAM tools were used to detect SNPs to ensure the accuracy of SNPs. SAM tools were used to remove duplicates and mask the effects of PCR duplication [45]. The obtained SNPs and small indels were annotated and predicted using SnpEff software [46], and only the high-quality SNPs with a minimum sequence read depth of five were used for BSA-Seq analysis.

### 4.3. Mapping of Candidate Genomic Regions by Association Analysis

Heterozygous and inconsistent SNPs (coverage depth >5×) between two contrasting F_2_ pools (AL and GL) were selected to calculate the Δ(SNP-index) values, and sliding-window analysis was performed for the association analysis. LOESS regression was performed for Δ(SNP-index) on the same chromosome to obtain the associated threshold. The candidate genomic regions were identified with an average *p*-value of *p* ≤ 0.01.

The Δ(SNP-index) of each locus was calculated with the formula, Δ(SNP-index) = Mindex–Windex, where m and n are ratios of accessions that exhibit the same bases as the hairy parents in the F2 hairy group and in the F2 glabrous group, respectively. A sliding-window analysis was applied to generate Δ(SNP-index) plots with a window size of 5 SNPs and increment of 2 SNPs. Significant high SNP-index values (the 0.1% in the right tail) were identified as the empirical thresholds, where the value is 0.525.

### 4.4. Gene Annotation in Candidate Regions

In order to annotate genes in candidate regions and analyze their functions, BLAST was used to compare the genes in the associated region with functional databases including NR [47], SwissProt [47], GO [48], COG [49] and KEGG [50].

### 4.5. RNA Isolation and qRT-PCR Analysis

The total RNA of W30, 082 and F2 with different phenotypes (trichome and glabrous leaves) was extracted using an RNAprep Pure Plant Kit (TianGen Biotech, Beijing, China). The quality and quantity of RNA were assessed using a Nanodrop 2000. Two micrograms of total RNA were reverse transcribed using Hifair^®®^ III 1st Strand cDNA Synthesis SuperMix for qPCR (gDNA digester plus) (Yeasen, Shanghai, China) following the recommended protocol. Products used as templates for qRT-PCR were diluted 5 times with ddH2O. Hieff^®®^ qPCR SYBR Green Master Mix (Yeasen, Shanghai, China) was employed to identify target gene expression, using a Fluorescent Quantity PCR Detectiong System (Bio-Rad I Cycler iQ5 Hercules, Foster City, CA, USA), in accordance with the manufacturer’s protocol. Relative gene transcript levels were determined using the method of the comparative threshold cycle (Ct) method with StepOne v.2.02 software installed in the real-time PCR system, and the 2-ΔΔCT method was used to measure the relative expression level measurement normalized to the internal control gene *BrActin* (*Bra028615*, [51]). The primer pairs were designed using GenScript (https://www.genscript.com/tools/real-time-pcrtaqman-primer-design-tool, accessed on 30 October 2020) and are listed in Table S3.

### 4.6. Statistical Analyses

We employed Tukey’s honestly significant difference (HSD) test to determine statistical significance. The difference was considered significant at *p* < 0.05.

## 5. Conclusions

In this study, three genes associated with leaf trichome formation were identified and verified in Chinese cabbage by sequencing-based bulked segregant analysis. As high-quality genomes have become more widely available, the BSA method has become an increasingly important tool for the rapid mapping of monogenic traits in diverse *Brassica* species. The results of this study suggest that BSA sequencing is valuable for the assisted-selective breeding of Chinese cabbage.

## Figures and Tables

**Table 1:** Genetic analysis of leaf trichome phenotype.

Generation	Trichome Leaves	Glabrous Leaves	Segregation Ratio
P1(W30)	40	0	
P2(082)	0	40	
F1	60	0	
F2	212	82	3:1

**Table 2:** Quality control of sequencing data for parental inbred lines W30 and 082 and bulked AL-pool and GL-pool samples.

Bulk	Clean Reads	Data Generated	Q30 (30%)	Genome Coverage (10×)	Average Depth (×)	SNP Number	Alignment Efficiency (%)
W30	37,948,696	11,384,608,800	92.89	93.43	27.5384	1,693,338	97
082	36,842,550	11,052,765,000	93.55	92.44	26.8289	1,663,130	97.26
AL	110,197,965	33,059,389,500	92.69	97.74	78.9208	1,740,465	97.28
GL	108,389,140	32,516,742,000	92.66	97.49	76.169	1,721,183	96.68

**Table 3:** SNP-select region information.

Chrom	Start	End	Length	Number of Genes
Scaffold000100	160,071	260,071	100,001	6
Scaffold001011	−49,220	50,780	100,001	0
Scaffold004266	−49,807	50,193	100,001	0
A06	22,044,767	22,246,745	201,979	30
A06	23,704,762	24,097,454	392,693	74
Scaffold000169	132,175	132,181	7	0

**Table 4:** Indel-select region information.

Chrom	Start	End	Length	Number of Genes
A07	1,892,096	1,992,096	100,001	13
A10	2,673,013	2,773,013	100,001	23
Scaffold000100	764,907	864,907	100,001	9

**Table 5:** Three genes related to trichome formation with function annotation by bulked segregant analysis (BSA).

Gene	Chr.	Function Annotation
*Bra025087*	A10	Cyclin family protein
*Bra035000*	Scaffold000100	ATP-binding/kinase/protein kinase/protein serine/threonine kinase
*Bra033370*	A06	WD-40 repeat family protein/beige-related

## Data Availability

The available data are presented in the manuscript.

## Supplementary Materials

The following are available online at https://www.mdpi.com/article/10.3390/plants10040771/s1, Figure S1. Sequence alignment of CYCT1;5 proteins in *Brassica rapa* and *Arabidopsis thaliana.* The white words with blue background represent *Brassica rapa* and *Arabidopsis thaliana* have the same amino acid. The black words with light blue background represent *Brassica rapa* and *Arabidopsis thaliana* have the different amino acids. Figure S2. Sequence alignment of NEK6 proteins in *Brassica rapa* and *Arabidopsis thaliana*. The white words with blue background represent *Brassica rapa* and *Arabidopsis thaliana* have the same amino acid. The black words with light blue background represent *Brassica rapa* and *Arabidopsis thaliana* have the different amino acids. Figure S3. Sequence alignment of SPI proteins in *Brassica rapa* and *Arabidopsis thaliana*. The white words with blue background represent *Brassica rapa* and *Arabidopsis thaliana* have the same amino acid. The black words with light blue background represent *Brassica rapa* and *Arabidopsis thaliana* have the different amino acids. Table S1. SNP-select region gene annotation, Table S2. Indel-select region gene annotation, Table S3 Primers used for qRT-PCR analysis.
