# Supplementary figures and images for: Mapping of Genetic Locus for Leaf Trichome Formation in Chinese Cabbage Based on Bulked Segregant Analysis
Source: Plants (Basel). 2021 Apr 14;10(4):771. doi: 10.3390/plants10040771 (PMC8070908; doi:10.3390/plants10040771)

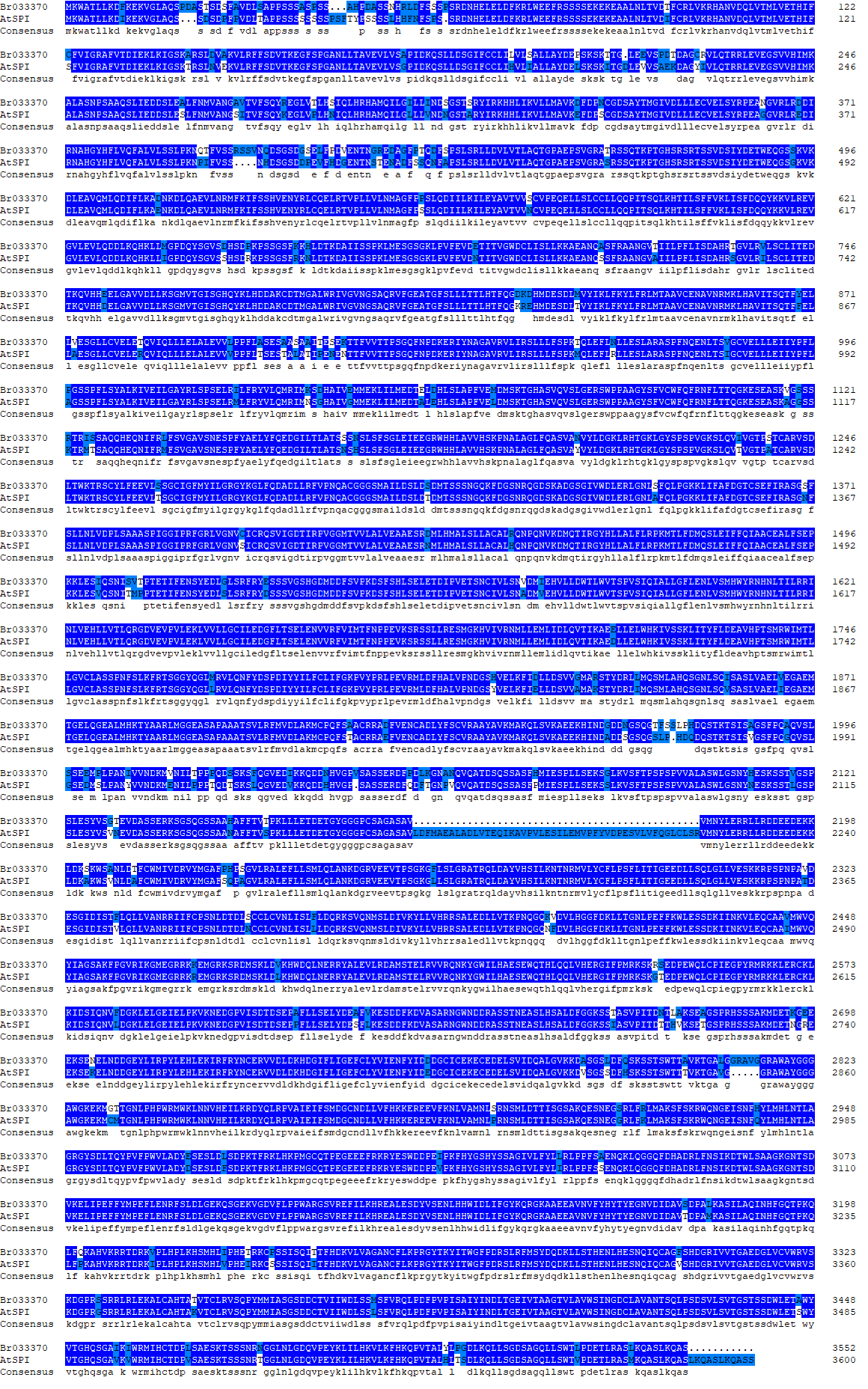

Supplement: Supplementary file 1 [file plants-10-00771-s001.zip › Figure S3. Sequence alignment of SPI proteins in Brassica rapa and Arabidopsis thaliana..tif]

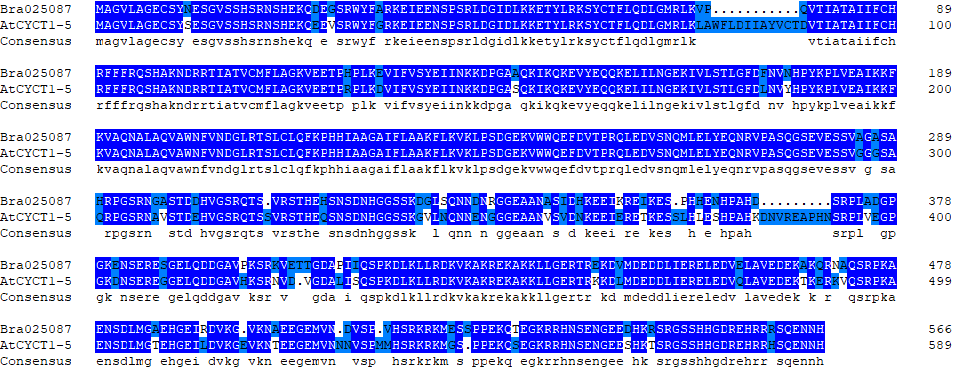

Supplement: Supplementary file 1 [file plants-10-00771-s001.zip › Figure S1. Sequence alignment of CYCT1;5 proteins in Brassica rapa and Arabidopsis thaliana..tif]

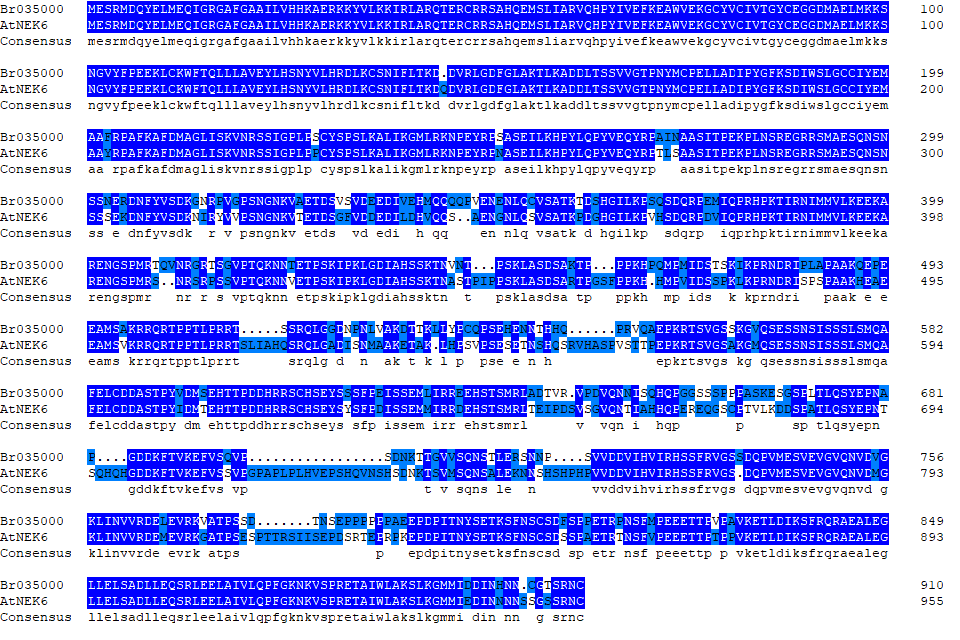

Supplement: Supplementary file 1 [file plants-10-00771-s001.zip › Figure S2. Sequence alignment of NEK6 proteins in Brassica rapa and Arabidopsis thaliana..tif]
